# The Multifaceted Role of Calcium Signaling in Regulated Necrosis

**DOI:** 10.3390/biom15060854

**Published:** 2025-06-11

**Authors:** Eric Perez-Rivera, Claudia Plasencia, Uris Ros

**Affiliations:** 1 Max Planck Institute of Biophysics, Max-von-Laue-Straße 3, 60438 Frankfurt am Main, Germany; eric.perez-rivera@biophys.mpg.de (E.P.-R.); claudia.plasencia@biophys.mpg.de (C.P.); 2 International Max Planck Research School (IMPRS) on Cellular Biophysics, Max Planck Institute of Biophysics, Max-von-Laue-Straße 3, 60438 Frankfurt am Main, Germany

**Keywords:** calcium signaling, cell death, necrosis, membrane damage, immune response

## Abstract

Calcium is a versatile ion that regulates diverse intracellular processes, including cell death and survival, cytokine and chemokine production, lipid scrambling, and immune cell activation. In regulated necrosis, an early increase in cytosolic calcium is a hallmark of pathways such as pyroptosis, necroptosis, and ferroptosis, and resembles the calcium surge triggered by pore-forming toxins. The complexity of calcium signaling is orchestrated by specialized channels in various cellular compartments and calcium-binding proteins that respond to localized calcium concentrations. However, the coordination of this intricate code during regulated necrosis and its connections to other calcium-driven processes remains poorly understood. This review provides an overview of the molecular mechanisms of calcium signaling in regulated necrosis, analyzing parallels with pore-forming toxin-mediated membrane damage to uncover nodes that are shared by these seemingly independent pathways. We also discuss advanced techniques for studying calcium dynamics, with high precision, that can be applied to study regulated necrosis. Calcium signaling emerges as a central hub where necrotic cell death pathways converge, shaping the unique signatures of dying cells and influencing their communication with the immune system. This integrated perspective highlights the complex and multifaceted role of calcium in cells and its implications for fundamental cellular processes.

## 1. Introduction

The plasma membrane acts as a vital barrier that protects cells by separating the intracellular environment from the extracellular space [1]. However, endogenous agents that form part of the intrinsic machinery responsible for necrotic forms of regulated cell death can compromise this vital function. During regulated necrosis, plasma membrane disruption is mediated by specialized proteins and lipids. For instance, pore-forming proteins (PFPs) such as gasdermins in pyroptosis and mixed lineage kinase domain-like protein (MLKL) in necroptosis are key players [2,3]. Similarly, lipids have a central role in ferroptosis-mediated membrane damage [4]. This process is similar to membrane damage mediated by pore-forming toxins (PFTs), which are a dedicated family of proteins that form part of the defense and attack mechanisms of bacteria and eukaryotes [5]. Due to the loss of plasma membrane integrity, these forms of necrosis, whether accidental or regulated, have an inflammatory phenotype with significant implications for inflammatory diseases such as atherosclerosis, autoimmune disorders, neurodegenerative diseases, and cancer [6,7].

Early calcium elevation is a hallmark of various types of regulated necrosis, resembling the calcium surge that occurs during membrane damage caused by PFTs [8,9,10]. Calcium is a versatile ion that plays a central role in cell signaling, mediating a wide array of cellular processes [11,12,13]. This includes not only cell death and survival but also cytokine and chemokine production, lipid scrambling, cell migration, and immune system activation. The coordination of these diverse processes relies on precise control mechanisms, including allosteric regulation, protein–protein interactions, and sub-cellular compartmentalization [13,14]. Specialized calcium signaling machinery comprises endogenous calcium channels located in different organelles and a broad set of calcium-binding proteins (CaBPs) [12,15]. These components work together to decode calcium signals by responding dynamically to variations in local calcium concentrations. This interplay ensures precise spatial and temporal control over calcium-dependent processes, enabling cells to coordinate complex biological functions with high specificity.

Despite significant progress, many questions remain regarding the role of calcium signaling in regulated necrosis. The precise mechanisms by which calcium regulates these distinct cell death pathways, including contributions from calcium dynamics in key organelles such as the plasma membrane, endoplasmic reticulum (ER), mitochondria, and lysosomes, are still unclear. Furthermore, the integration of calcium fluxes with plasma membrane permeabilization, which is mediated by endogenous pore-forming executioners, and the manner in which calcium facilitates processes such as membrane repair and lipid scrambling, which tip the balance between cell death and survival, remain poorly understood. Similarly, the temporal dynamics of calcium signaling and its interplay with immunogenicity, including the release of damage-associated molecular patterns (DAMPs), are yet to be elucidated.

In this review, we examine the molecular mechanisms of cell death and calcium signaling in various forms of regulated necrosis, including pyroptosis, necroptosis, and ferroptosis. We first provide an overview of these seemly independent cell pathways to dissect key components involved in the interconnected signaling. We extend this analysis to PFTs-mediated cell death as a parallel model to offer a unifying framework to understand calcium signaling following membrane damage. Furthermore, we discuss emerging topics under debate, including the role of calcium in cell death and survival, as well as in the immunity of dying cells. Finally, we summarize state-of-the art techniques that can be applied for studying calcium dynamics with high precision, in the context of regulated necrosis. We provide both a conceptual and a practical guide for researchers at the interface of calcium signaling and cell death fields.

## 2. The Molecular Mechanism of Necrotic Forms of Cell Death

**Figure 1:**
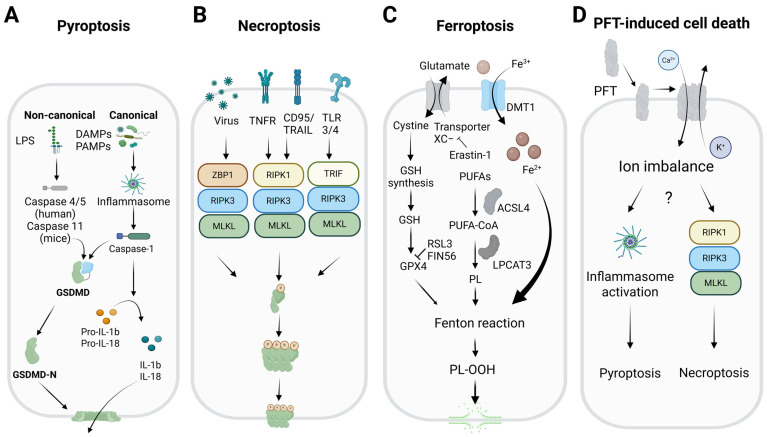
The molecular mechanisms of different necrotic forms of cell death. (**A**) Pyroptosis is mediated by inflammatory caspases (caspase-1 in the canonical inflammasome pathway and caspase-4/5 (in humans), or caspase-4 (in mice) in the non-canonical pathway). The inflammatory caspases cleave GSDMD to release its N-terminal domain with pore-forming activity. (**B**) Necroptosis is mediated by RHIM-domain-containing proteins (RIPK1, TRIF, and ZBP1), which bind and activate RIPK3, a kinase that phosphorylates and activates MLKL, the protein that permeabilizes the plasma membrane via a still unclear mechanism. (**C**) Ferroptosis is a unique form of necrotic cell death activated by an imbalance of the antioxidant system of the cell. GPX4 is a key enzyme in ferroptosis that reduces the levels of peroxidized phospholipids in membranes by converting them into less toxic forms, using glutathione as a co-substrate. Inactivation of this enzyme (e.g., by glutathione depletion or chemical inhibition) triggers ferroptosis. ACSL4 and LPCAT3 are also two key enzymes in ferroptosis, as they facilitate the incorporation of PUFAs into cellular membrane phospholipids. (**D**) PFTs are proteins produced by various organisms as part of their attack or defense mechanisms. They are synthetized as soluble monomers, and are able to interact with the outer leaflet of the plasma membrane, where they oligomerize to form pores that allow the passage of molecules and ions. At high concentrations, they induce irreversible membrane damage, leading to cell death by necrosis. However, in some contexts, they can activate regulated cell death pathways, such as necroptosis and pyroptosis. LPS: lipopolysaccharides, DAMPs/PAMPs: damage/pathogen associated molecular patterns, GSDMD: gasdermin D, N-terminal GSDMD (GSDMD-N), IL: interleukin, TNF: tumor necrosis factor, TRAIL: tumor necrosis factor-related apoptosis-inducing ligand, TLR: toll-like receptor, RHIM: RIP homotypic interaction motif, ZBP1: Z-DNA binding protein 1, RIPK1: receptor-interacting protein kinase 1, TRIF: TIR-domain-containing adapter-inducing interferon-β, RIPK3: receptor-interacting protein kinase 3, MLKL: mixed lineage kinase domain-like, DMT: divalent metal transporter, GSH: glutathione, GPX4: glutathione peroxidase 4, PUFAs: polyunsaturated fatty acids, ACSL4: acyl-CoA synthetase long chain family member 4, LPCAT3: lysophosphatidylcholine acyltransferase 3, PL-OOH: oxidized phospholipids, PFT: pore-forming toxin. Created with Biorender.com.

Necrosis was initially considered an accidental and uncontrolled form of cell death that occurred in response to overwhelming chemical or physical damage [6,7]. The main characteristic of this form of cell death is the rupture of the cell membrane, which causes the release of cellular contents into the extracellular space [7]. These molecules are recognized by immune cells, triggering an inflammatory response [16]. More recent evidence has shown that necrosis can also occur as a programmed process, regulated by different mechanisms [6]. The most well-studied forms of regulated necrosis are pyroptosis, necroptosis, and ferroptosis (Figure 1). Conversely, proteins belonging to the PFT family are key mediators of plasma membrane permeabilization, particularly during bacterial infections [17,18]. These proteins can induce cell death through mechanisms that overlap with both accidental and regulated necrosis [19,20]. This section provides an overview of the underlying mechanisms of these different forms of necrotic cell death.

### 2.1. Pyroptosis

Pyroptosis is a pro-inflammatory, caspase-dependent form of regulated cell death associated with innate immunity, which is triggered by intracellular danger signals or external pathogens (Figure 1A). It is characterized by morphological changes such as chromatin condensation, cell swelling, and plasma membrane rupture, and it is distinct from apoptosis [21]. It is mainly activated through two pathways: the canonical and the non-canonical. In the canonical pathway, pathogen- or damage-associated molecular pattern (PAMP or DAMP) receptors such as nucleotide-binding oligomerization domain (NOD) receptors (NLRPs) and absent in melanoma 2 (AIM2), detect the stimuli and activate the inflammasome, a protein complex that recruits and activates caspase-1 [22,23,24]. In the non-canonical pathway, human caspase-4/5 or murine caspase-11 are activated by intracellular bacterial lipopolysaccharides (LPS), which activate the NLRP3 inflammasome, linking the two pathways to intensify the inflammatory response [22,25,26]. In both pathways, the inflammatory caspases process gasdermin D (GSDMD) and pro-inflammatory cytokines such as interleukin (IL)-1β and IL-18 [27,28,29]. The N-terminal portion of GSDMD (GSDMD-N) forms pores in the plasma membrane, allowing the release of cytokines and other cellular contents. The dual role of GSDMD and the pro-inflammatory potential of pyroptosis highlight its importance in the immune response and its involvement in chronic inflammatory diseases [30].

### 2.2. Necroptosis

Necroptosis is a regulated form of lytic cell death, triggered in conditions of caspase-8 inactivation where apoptosis is inhibited (Figure 1B) [31,32]. This process is initiated by alterations in the cellular microenvironment that are detected by specific receptors, such as the death receptors (DRs), including tumor necrosis factor receptor-1 (TNFR1), tumor necrosis factor-related apoptosis-inducing ligand (TRAIL), and Fas receptor (CD95); toll-like receptors (TLRs) such as TLR3/4; or the protein Z-DNA-binding protein 1 (ZBP1), which recognizes DNA or RNA in a Z conformation [33,34,35,36]. When the TNFR-mediated pathway is activated, and cellular inhibitor of apoptosis proteins (cIAPs) are inhibited, the receptor-interacting serine/threonine-protein kinase 1 (RIPK1) interacts with the Fas-associated death domain (FADD) and caspase-8, forming a complex that leads to apoptosis [37]. If caspase-8 is absent or inhibited, RIPK1 forms an alternative complex, the canonical necrosome, by binding to RIPK3 [38]. Necroptosis can also be initiated by TLR3/4 activation via TRIF or by ZBP1 binding to Z-DNA or Z-RNA. This allows RIPK3 to form a non-canonical necrosome via interactions with the RIP homotypic interaction motif (RHIM) [33,38]. In both types of necrosome, RIPK3 is activated by autophosphorylation, leading to the phosphorylation and subsequent activation of MLKL [39]. Afterward, MLKL oligomerizes and translocates to the plasma membrane to mediate its permeabilization and the release of the intracellular contents [3]. Despite its relevance, the precise mechanisms by which MLKL causes cell death are still not fully understood.

### 2.3. Ferroptosis

Ferroptosis is a special form of regulated necrosis that does not rely on traditional signaling pathways but instead on the deregulation of the antioxidant system of the cell. In this case, it is the iron-dependent accumulation of lipid peroxides that leads to the disruption of membrane integrity (Figure 1C) [4,40]. This process is primarily regulated by glutathione peroxidase 4 (GPX4), a key enzyme that reduces the toxicity of peroxidized phospholipids in membranes by converting them into less toxic forms, using glutathione as a co-substrate [41]. Ferroptosis inducers (FINs) are classified into four categories: (1) compounds such as erastin-1, which inhibit the Xc^-^ transporter and deplete glutathione levels [42]; (2) molecules such as RAS-selective lethal small molecule 3 (RSL3), which directly inactivate GPX4 [43]; (3) FIN56, which promotes GPX4 degradation and Coenzyme Q10 (CoQ10) depletion [44]; and (4) FINO2, which induces iron oxidation and lipid peroxidation, although its mechanism is unclear [45]. Ferroptosis can also be induced by intrinsic pathways involving the regulation of transporters and antioxidant enzymes, as well as extrinsic pathways that suppress the expression or activity of these enzymes [4]. Besides GPX4, other inactivators of ferroptosis include ferroptosis suppressor protein 1 (FSP1), CoQ10-nicotinamide adenine dinucleotide phosphate (NADPH), and tetrahydrobiopterin (BH4) [46,47,48]. The metabolism of polyunsaturated fatty acids (PUFAs) is closely related to ferroptosis. Two key enzymes that facilitate the incorporation of PUFAs into cellular membrane phospholipids are long-chain acyl-CoA synthetase (ACSL4) and lysophosphatidylcholine acyltransferase 3 (LPCAT3) [49,50]. Inhibiting ACSL4 protects cells from ferroptosis, whereas the effect of inhibiting LPCAT3 is less significant [49,50]. Iron transport and the regulation of glutaminolysis also play an important role in ferroptosis. Transferrin and its cellular receptor are involved in cysteine deprivation and iron accumulation, which triggers lipid peroxidation [51]. It has also been suggested that proteins such as the mitochondrial outer membrane [2Fe-2S] protein (mitoNEET), which regulates mitochondrial iron transport, may play a role in ferroptosis [52]. Ferroptosis is therefore a complex mechanism of cell death involving multiple antioxidant and lipid-regulatory pathways.

### 2.4. Pore-Forming Toxins

PFTs are produced by various organisms as part of their attack or defense mechanisms and are among the most potent virulence factors found in nature [17]. Examples include α-toxin from *Clostridium perfringens* [53], members of the cholesterol-dependent protein family (e.g., lysozyme O from *Listeria monocytogenes*, aerolysin from *Aeromonas hydrophila*) [54], and actinoporins (e.g., sticholysins and fragaceatoxin) from sea anemones [55]. Like the executioners of regulated necrosis (e.g., gasdermins and MLKL), these proteins are produced as soluble monomers, able to interact with the plasma membrane, specifically the outer leaflet (Figure 1D) [54,55]. Once at the membrane, they oligomerize to form pores that allow the passage of molecules and ions, thereby leading to disturbances in the ionic balance of the cytoplasm [5]. At high concentrations, PFTs induce irreversible membrane damage, leading to cell death by necrosis. During this process, the cell swells and loses its boundaries, ultimately dying [17,18]. This process is often accompanied by the formation of blebs, which are globular protrusions that appear on the cell surface following significant membrane damage. These blebs probably serve to isolate and expel the toxin, or act as liquid reservoirs to help alleviate the increase in cell volume [56]. In some contexts, certain PFT family members can activate regulated cell death pathways, such as necroptosis and pyroptosis [17,19]. For example, caspases-4 and -11 mediate epithelial defenses against enteric bacterial pathogens by activating inflammasomes [57]. Additionally, PFTs can induce necroptosis in macrophages and respiratory epithelial cells, contributing to lung damage during bacterial pneumonia [58]. Interestingly, PFT-induced necroptosis occurs independently of death receptor activation and is instead associated with ionic dysfunctions resulting from membrane permeabilization.

## 3. Channels and Proteins Involved in Calcium Signaling and Their Functions

**Figure 2:**
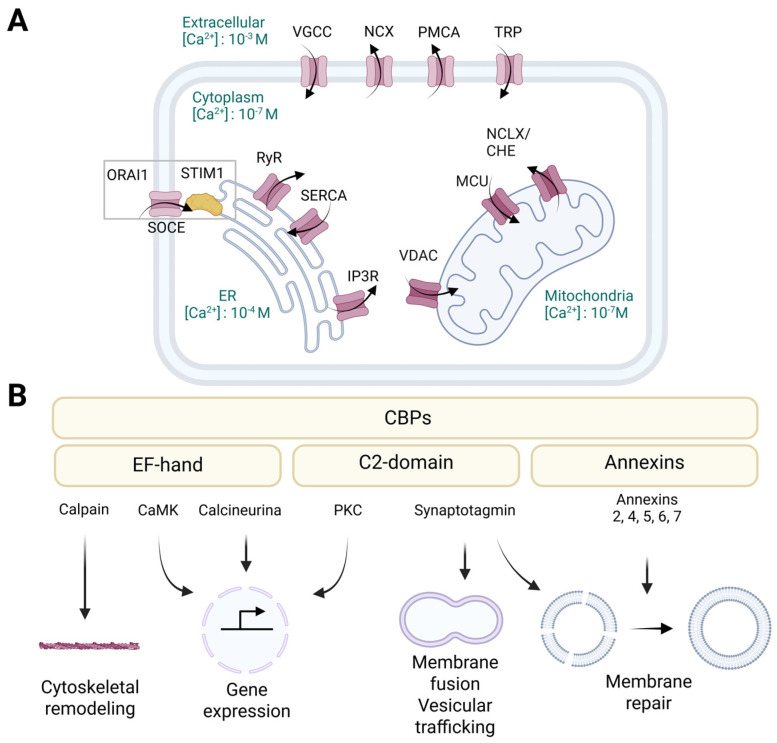
The cellular machinery that mediates calcium signaling. (**A**) Different calcium channels located at the plasma membrane or intracellular organelles facilitate calcium entry and fluxes within the cell. Plasma membrane channels include the VGCCs and the TRP, which mediate the entry of calcium into the cytosol, and the PMCA and NCX, which pump calcium out of the cell to return to resting levels. The ER and mitochondria are the main intracellular calcium reservoirs. SOCE, involving STIM1 and ORAI1, becomes active when ER calcium stores are depleted, promoting the influx of extracellular calcium to restore internal levels. SERCA is a high-affinity pump that continuously replenishes ER calcium levels by taking up calcium into the ER. Calcium is released from the ER via RyRs or IP_3_Rs. VDAC and MCU mediate calcium influx to the mitochondria, while NCLX mediates calcium exit. (**B**) The family of CaBPs bind calcium through specific structural motifs and have a common function in transducing calcium signaling in cells. Depending on the structural motif that binds to calcium, they can be classified as EF-hand proteins, annexins, or C2-domain proteins. They can act as calcium buffers or regulators of various cellular processes such as gene expression, signal transduction, vesicular trafficking, cytoskeleton remodeling and membrane repair. VGCCs: voltage-gated calcium channels, NCX: Na^+^/Ca^2+^ exchanger, PMCA: plasma membrane Ca^2+^ ATPase, TRP: transient receptor potential channels, ORAI1: calcium release-activated calcium channel protein 1, SOCE: store-operated calcium entry, STIM1: stromal interaction molecule 1, RyRs: ryanodine receptors, SERCA: sarcoplasmic/endoplasmic reticulum calcium ATPase, IP_3_Rs: inositol 1,4,5-trisphosphate receptors, ER: endoplasmic reticulum, VDAC: voltage-dependent anion channel, MCU: mitochondrial calcium uniporter, NCLX: sodium/calcium/lithium exchanger, CaBPs: calcium-binding proteins, CaMK: calcium/calmodulin-dependent kinases, PKC: protein kinase C. Created with Biorender.com.

Both regulated necrosis (pyroptosis, necroptosis, ferroptosis) and PFT-induced cell death involve the permeabilization of the plasma membrane, leading to an increase in the concentration of cytosolic calcium [59]. Calcium acts as a secondary messenger in various cellular processes, and disturbances in calcium homeostasis can trigger diverse signaling cascades that ultimately determine cell fate (Figure 2) [12,13,60]. Given this critical role, it is essential to dissect the molecular interplay between calcium influx and membrane disruption. In this section, we provide an overview of the endogenous cellular machinery responsible for calcium transport and signaling. This summary facilitates further analysis of how the complex calcium machinery intersects with cell death pathways as part of the unified cellular response to membrane damage.

To prevent calcium-induced cytotoxic damage, the level of calcium in the cells is carefully controlled through mechanisms that regulate its influx, efflux, storage, and buffering mechanisms, collectively known as calcium homeostasis [12]. Within cells, calcium exists primarily in three main forms: storage, conjugated, and free calcium. In resting states, free cytosolic calcium is kept at around 100 nM, which is approximately 10,000 times lower than its extracellular concentration. This steep gradient is critical for enabling rapid calcium signaling upon stimulation. Intracellular calcium homeostasis is primarily regulated by influx from the extracellular environment via the plasma membrane and the release from internal stores, mostly in the ER and mitochondria (Figure 2A) [12,15].

Calcium signals exhibit both temporal and spatial specificity [61]. Temporary calcium spikes or oscillations can occur when intracellular calcium levels quickly increase from 100 nM to more than 1 µM. These dynamic changes are regulated by the coordinated opening and closing of calcium channels, as well as feedback mechanisms involving pumps and exchangers [62]. Different cellular responses are initiated based on the amplitude, frequency, and duration of calcium fluctuations, highlighting the sophistication and precision of this signaling system [12,62]. Ultimately, maintaining calcium homeostasis under typical physiological circumstances depends on an intricate web of calcium channels, pumps, exchangers, and sensors that work together to precisely control intracellular calcium levels.

### 3.1. Calcium Channels and Transporters

Various transport systems within the plasma membrane facilitate the entry and removal of calcium (Figure 2A). Voltage-gated calcium channels (VGCCs), which are predominantly active in excitable cells such as neurons and muscle cells, open upon depolarization, allowing quick calcium influx [63]. Transient receptor potential (TRP) channels respond to mechanical, chemical, and thermal stimuli to enhance calcium entry [64]. Store-operated calcium entry (SOCE), which involves the stromal interaction molecule 1 (STIM1) and the calcium release-activated calcium modulator 1 (ORAI1), becomes active when ER calcium stores are depleted, promoting the influx of extracellular calcium to restore internal levels [65]. The plasma membrane calcium ATPase (PMCA) and the sodium/calcium exchanger (NCX) assist in removing calcium from the cytoplasm and returning it to resting levels [66]. Moreover, the calcium-sensing receptor (CaSR), which is a G-protein-linked receptor, regulates systemic calcium levels in response to external calcium concentrations [66].

The ER serves as the primary intracellular calcium reservoir, with concentrations varying from 100 μM to 1 mM (Figure 2A) [67]. The sarcoplasmic/endoplasmic reticulum calcium ATPase (SERCA), a high-affinity pump, continuously replenishes ER calcium levels by taking up calcium into the ER [13,67,68]. Conversely, calcium is released from the ER through ryanodine receptors (RyRs) and inositol 1,4,5-trisphosphate receptors (IP_3_Rs), which are triggered by signaling molecules such as inositol 1,4,5-trisphosphate (IP_3_) or changes in membrane potential [13,67]. Additionally, basal calcium leakage is assisted by calcium leak channels in the ER [69]. Excessive calcium loss or accumulation can result in ER stress and disrupt protein folding [67].

Mitochondria play a significant role in maintaining intracellular calcium homeostasis, acting as essential sites for calcium buffering and signal integration (Figure 2A) [70,71]. Calcium influx into the mitochondria is mediated by the voltage-dependent anion channel (VDAC) and the mitochondrial calcium uniporter (MCU), which allow the entry of calcium into the mitochondrial matrix, particularly during surges in cytosolic calcium [68]. Calcium loading into the mitochondria can activate metabolic enzymes involved in the tricarboxylic acid (TCA) cycle, thereby linking calcium signaling to energy production [71]. In contrast, calcium exit is mediated by the sodium/calcium/lithium exchanger (NCLX) and the calcium/hydrogen exchanger (CHE), which prevent mitochondrial calcium overload that could impair function and induce cell death [70,72]. Notably, the connections between the ER and mitochondria at the mitochondria-associated ER membranes (MAMs) facilitate rapid and localized calcium transfer between these organelles, impacting metabolic regulation and triggering apoptosis [68].

### 3.2. Calcium Sensing Proteins

The CaBPs protein family comprises diverse proteins that can bind calcium through specific structural motifs (Figure 2B) [12]. Depending on the shared structural motif that binds calcium, they can be classified as EF-hand proteins (e.g., calmodulin (CaM), calcineurin, calpain, calcium/calmodulin-dependent kinases (CaMK), and parvalbumin), annexins, and C2-domain proteins (e.g., synaptotagmins and protein kinase C (PKC)) [12,73]. The EF-hand calcium-binding motif consists of a calcium-coordinated loop flanked by two nearly perpendicular α-helices. The calcium ion is coordinated by seven ligands, predominantly carboxylate groups, in a pentagonal bipyramidal configuration [74]. In the annexins and C2-domain proteins, specialized calcium-binding regions enable binding to negatively charged membranes, thus connecting membrane functions with calcium signaling [75,76]. Annexins differ from EF-hand motifs in that they coordinate calcium with five oxygen atoms derived from proteins [76]. Conversely, C2-domains utilize a β-sheet core to bind calcium and phospholipids rather than the α-helical structure of annexins. C2-domains exhibit different sensitivities to lipids and calcium levels due to variations in the loops that connect the β-sheets [75].

Members of the CaBP family share a unique function in sensing and integrating calcium signaling in cells [12,73]. They can serve either as calcium buffers or regulators of various cellular processes, including signal transduction, muscle contraction, enzyme activity, cytoskeleton remodeling and membrane trafficking (Figure 2B) [12]. EF-hand domains represent the most prevalent calcium-binding motifs in proteins [73,74]. This group of proteins performs various functions, such as regulating gene expression in the nucleus, mediating signal transduction between compartments, and buffering calcium levels in the cytoplasm [77]. As calcium binds to EF-hand domains with varying affinities (ranging from 10^−6^ M to 10^−3^ M), these proteins exhibit diverse biological activities at different calcium concentrations [74,77]. For example, the phosphatase activity of calcineurin can be affected by both direct calcium interaction or via CaM [78]. Upon activation, calcineurin stimulates transcription factors such as the nuclear factor of activated T cells (NFAT), thereby activating the immune response [78]. Conversely, calpains are solely regulated by direct calcium interaction with their EF-hand domains [79]. CaMKs are another type of EF-hand protein that transduce the intracellular calcium signals into changes in the phosphorylation state and activity of target proteins [80]. The capacity of CaMK to trap CaM enables these enzymes to detect the frequency of the calcium signals [80,81]. Depending on their downstream targets, these proteins can be divided into multifunctional kinases, which affect multiple processes, and substrate-specific kinases, which usually have a specific function within the cell or tissue in which they are expressed [80]. On the other hand, annexins act as membrane scaffolding proteins, anchoring other proteins to the cell membrane and organizing lipid microdomains [82]. They facilitate membrane curvature, vesicle budding, and trafficking events such as endocytosis and exocytosis [83]. For instance, annexin 2 organizes lipid rafts and interacts with actin to support membrane-cytoskeleton dynamics [84]. In addition, upon plasma membrane injury and subsequent calcium influx, annexins rapidly accumulate at wound sites to aid membrane repair [83]. Another group of CaBPs involved in membrane-related processes are the synaptotagmins, which mediate membrane fusion and play a key role in neurotransmission at synapses, as well as regulating intracellular membrane trafficking pathways [85]. For instance, synaptotagmin 17 (SYT7) is localized to the Golgi complex, where it coordinates the import of vesicles from the ER into the Golgi by physically interacting with key Golgi proteins, which are involved in tethering and importing cargo from the ER [86].

Despite substantial progress in understanding calcium signaling as an integrated mechanism for maintaining cell homeostasis, there are still significant gaps in our knowledge of how calcium channels and CaBPs influence the outcome of lytic cell death. It is unknown whether diverse membrane insults converge on shared calcium-dependent signaling pathways. An open question is how specific CaBPs such as annexins or synaptotagmins interpret these calcium fluxes to mediate cell-type-specific or context-dependent outcomes involving membrane repair, cytoskeletal rearrangements, mitochondrial dysfunction, or secretion of inflammatory factors. In order to clarify how calcium signaling integrates lytic cell death and inflammation, it is essential to identify shared molecular nodes and effector mechanisms. In the following sections, we review current knowledge of calcium signaling in regulated necrosis, aiming to integrate these two seemingly independent cellular processes.

## 4. The Complex Code of Calcium Signaling During Regulated Necrosis: What We Know So Far

The source of calcium fluxes during regulated necrosis is the subject of ongoing debate, as they may arise either directly through protein-mediated pores in the plasma membrane or indirectly via the activation of endogenous channels. Nevertheless, it is widely accepted that extracellular calcium plays a critical role in regulated necrosis, as calcium-free environmental conditions effectively inhibit cell death [8,9,59]. In line with the direct model, it has been proposed that calcium influx can occur through gasdermin- or MLKL-formed pores, in pyroptosis and necroptosis, respectively [8,87,88]. However, there is still a lack of direct evidence demonstrating the capacity of these two proteins to mediate calcium transport across membranes. Moreover, endogenous calcium channels within intracellular organelles may amplify signals triggered by membrane damage during regulated necrosis (Table 1). In this section, we provide an overview of how extracellular and intracellular calcium contribute to different forms of regulated necrosis and point out some parallels with PFT-induced cell death.

In pyroptosis, an increase in cytosolic calcium takes place before total membrane permeabilization via a process that is thought to be mediated by GSDMD pores [9,88]. In support of this, optogenetic activation of GSDMD induced a gradual increase in whole-cell cytosolic calcium through a mechanism involving localized calcium spikes, a process that was inhibited in calcium-free media [88]. Furthermore, in LPS-induced pyroptosis, GSDMD deficiency protected against plasma membrane rupture and abolished the early calcium influx [9]. These results suggest that extracellular calcium is the main source of the characteristic increase in cytosolic calcium during pyroptosis. Interestingly, elevated extracellular calcium can also activate the NLRP3 inflammasome in monocytes and macrophages via G protein-coupled calcium-sensing receptors (CaSR and GPRC6A) [90]. This also leads to the activation of the phosphatidylinositol/calcium signaling pathway, resulting in increased intracellular calcium, inflammasome assembly, and caspase-1 activation [90]. However, in pyroptosis induced by *Salmonella* infection or ATP treatment (LPS primed), the secretion of the caspase-1 substrates IL-18 and IL-1β occurs independently of extracellular calcium [100]. Recent studies have demonstrated that intracellular calcium release from the ER, particularly via the IP_3_R2 receptor, can promote pyroptosis by activating the NLRP3/caspase-1/GSDMD pathway, particularly in response to LPS stimulation [89]. Moreover, phospholipase C (PLC) γ1-mediated calcium signaling enhances the translocation of GSDMD-N to the plasma membrane, thereby amplifying pyroptotic cell death. These studies indicate that both the influx of calcium from outside the cell and its mobilization from inside are important for optimal execution of pyroptosis [91].

In necroptosis, the role of calcium varies considerably across different cellular systems [31]. In a study in which necroptosis was induced in mouse fibroblasts using TNF, Smac mimetic, and a pan-caspase inhibitor (zVAD) (TSZ), two distinct waves of calcium were identified [8]. The initial rise in calcium was primarily attributed to the Smac mimetic LCL-161, as it occurred independently of necrosome formation or MLKL activation. Conversely, the second wave appeared later, correlating with plasma membrane permeabilization and relying on RIPK3 and MLKL [8]. Importantly, this second and specific wave was conserved upon activation of TLR3/4-mediated necroptosis in the absence of the Smac mimetic [8]. Supporting the central role of MLKL in mediating calcium fluxes during necroptosis, cytosolic calcium elevation is abolished in MLKL knockout cells, whereas direct chemical or optogenetic activation of MLKL triggers it [3,87]. However, alternative models propose that MLKL oligomers form cation channels that are permeable to magnesium, sodium, and potassium, but not calcium. This opens up the possibility that the calcium elevation observed during necroptosis could result from the secondary effects of membrane damage and ionic imbalance [101,102]. It was proposed that MLKL may interact with or cause conformational changes in endogenous calcium channels like transient receptor potential melastatin-subfamily member 7 (TRPM7) [92]. This potential interaction could explain the increase in cytosolic calcium observed in necroptosis, even if MLKL is not a calcium channel. Further evidence suggests that RIPK3 can activate CaMK type 2 delta (CAMK2D), independently of MLKL, leading to ion influx through various ion channels [103]. Yet, there is limited evidence regarding the contribution of intracellular calcium to necroptosis, although some studies suggest that alongside extracellular influx, release from intracellular stores can also play a role. Experiments using the extracellular calcium chelator EGTA or the intracellular calcium chelator BAPTA-AM showed that, at least in mouse fibroblasts, calcium comes from both sources [8]. Supporting this, the downregulation of STIM1 and ORAI1 hindered loss of plasma membrane integrity in necroptosis [93].

In ferroptosis, it has been demonstrated at a single cell level that an increase in cytosolic calcium typically precedes complete cell bursting [10]. However, as with necroptosis, the contribution, timing, and amplitude of calcium fluxes can vary depending on the cell type and the inducer used. For example, erastin-1 or RSL3 treatments can result in different patterns and magnitudes of calcium influx. While RSL3 induces a distinctive calcium increase that can be linked to pore formation, erastin-1 triggers two calcium spike events. The first, non-specific spike appears to be related to the activation of VDAC channels, while the second spike is related to pore formation [10]. Early studies on oxytosis (which is similar to ferroptosis) demonstrated that eliminating extracellular calcium inhibits cell death, leading to the initial hypothesis that the elevation of cytosolic calcium in ferroptosis results from extracellular influx [104,105]. Recent research emphasizes the crucial role of intracellular calcium stores in regulating ferroptosis [59,105]. In line with this, the broad inhibition of calcium channels using cobalt chloride (CoCl_2_) has demonstrated protective effects against ferroptosis induced by erastin-1 or RSL3, suggesting that calcium fluxes from the ER or mitochondria could be essential for this type of cell death [105]. Conversely, increased ER calcium levels make cells more susceptible to ferroptotic death, emphasizing the important function of this organelle as a calcium reservoir that affects sensitivity to lipid peroxidation. Indeed, genetic downregulation of ORAI1 and ORAI3 provided protection against ferroptosis [97]. In neuroblastoma cells treated with RSL3, calcium signals were inhibited by blocking the IP_3_R with xestospongin B, reducing IP_3_R levels with carbachol, or knocking down IP_3_R1 [95]. These interventions also prevent RSL3-induced morphological changes, such as cell rounding. Different studies have proposed that mitochondrial calcium uptake also contributes to the regulation of ferroptosis. Consistent with this, the mitochondrial calcium uptake 1 (MICU1) is essential for effective lipid peroxidation during ferroptosis induced by cold stress, indicating that mitochondrial calcium influx can amplify ferroptotic cell death under particular conditions [96]. Overall, these findings suggest that intracellular calcium stores, particularly those in the ER and mitochondria, play a crucial role in ferroptosis.

**Figure 3:**
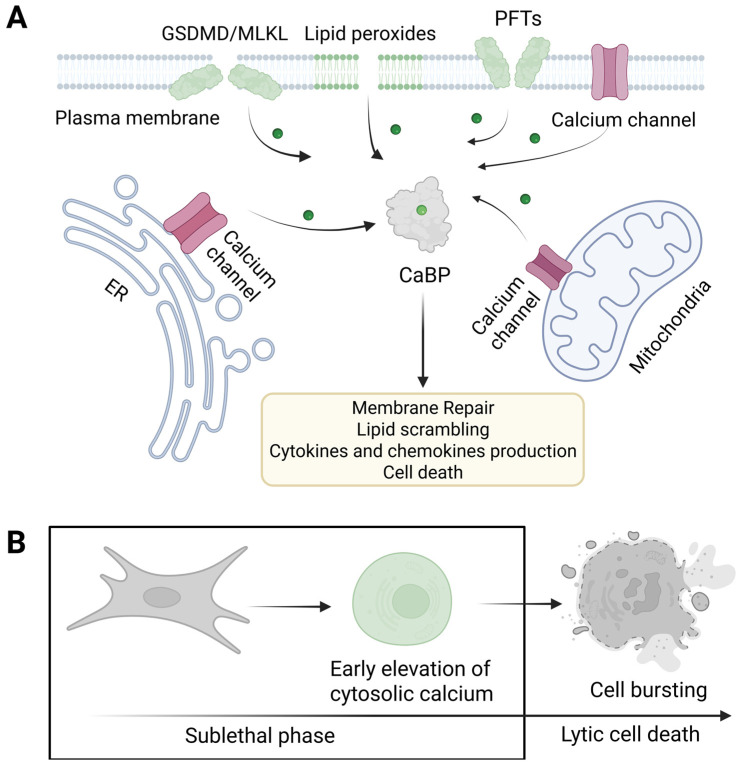
Membrane pores cooperate with endogenous calcium channels to trigger calcium elevation during necrotic cell death. (**A**) PFTs form pores in the outer leaflet of the plasma membrane, whereas endogenous executioners of regulated necrosis form permeabilize the cellular membrane through their action in the inner leaflet. Influx of cytosolic calcium can take place via plasma membrane pores or endogenous calcium channels, located either in the plasma membrane or intracellular organelles, namely ER and mitochondria. Endogenous calcium channels and CaBPs would cooperate with the amplification of the signaling initiated by pore formation to mediate various calcium-related processes such as membrane repair, lipid scrambling, the production of cytokines, and ultimately cell death. (**B**) The delay between the initial membrane injury, calcium elevation, and final cell lysis represents the time window in which different calcium-dependent processes can occur during the sublethal phase preceding cell lysis. GSDMD: gasdermin D, MLKL: mixed lineage kinase domain-like, PFTs: pore-forming toxins, ER: endoplasmic reticulum, CaBPs: calcium-binding proteins. Created with Biorender.com.

Although it is well established that calcium, whether released from intracellular stores or entering from the extracellular space, plays a key role in different forms of regulated necrosis, the specific contributions of each source remain unclear. Is intracellular calcium essential for committing cells to death, or is the influx from extracellular media sufficient? Does calcium efflux from organelles such as the ER or mitochondria precede plasma membrane disruption? If so, is there a coordinated interplay between these events? What are their biological functions? Furthermore, while initial progress has been made in identifying endogenous calcium channels and transporters involved in regulated necrosis, the synergy or redundancy of the different molecular players remains largely unknown (Figure 3A). Systematic functional screening is required to determine which channels and transporters actively mediate calcium dynamics during regulated necrosis. Clarifying these mechanisms is critical to understand how calcium signaling integrates membrane damage with the execution and regulation of necrotic cell death.

### Increase in Cytosolic Calcium as a Conserved Mechanism Downstream of Membrane Damage: What We Learn from PFTs

An increase in cytosolic calcium is a well-conserved mechanism following membrane damage, and it is particularly important in orchestrating the cellular response to both regulated necrosis and PFTs (Figure 3) [5,18,20]. It has long been thought that extracellular calcium is the primary source of calcium influx following PFT-mediated membrane injury, as these proteins create pores in the plasma membrane that can potentially allow substantial calcium entry, driven by the steep concentration gradient between the extracellular space and the cytosol [5]. However, the pattern of calcium influx triggered by different PFTs would vary depending on the selectivity of the pore, which is influenced by both the size and charge distribution in the lumen [5]. Therefore, the ability of a PFT to mediate calcium fluxes is determined by the specific characteristics of its pores, their interactions with membrane lipids or receptors, as well as their concentration and the host cell’s repair efficiency [20].

A monophasic influx of calcium is characterized by an immediate, and sustained increase in cytosolic levels. This typically occurs when pores are stable and the membrane repair processes are either overwhelmed or ineffective [20]. Conversely, a multiphasic influx features oscillatory or repetitive spikes of calcium entry, which often indicate cycles of pore formation and active membrane repair. When the cell can partially repair the membrane or eliminate pores (for example, via endosomal sorting complexes required for transport (ESCRT)-III or endocytic processes), calcium entry may temporarily decrease, only to increase again if additional pores develop or existing ones remain [20]. The different influx modes can lead to a variety of cellular responses, ranging from activating membrane repair to mechanisms inducing cell death [19,58,106]. The specific outcome would depend on the severity and duration of calcium overload, alongside the unique properties of the pore [20]. While these different mechanisms have been described for the action of PFTs, the way in which they are coordinated during regulated necrosis remains unclear. Determining whether regulated necrosis follows similar calcium dynamics or if it involves distinct spatial or temporal signatures could help distinguish between reversible damage and commitment to cell death. To address these questions, future studies should combine live-cell calcium imaging with genetic and pharmacological manipulation of endogenous PFPs and repair pathways, ideally in parallel with cell fate and inflammatory output measurements.

Experimental evidence demonstrates that chelating extracellular calcium protects cells from PFT-induced death, underscoring the centrality of calcium overload in this process [107]. In addition, different PFTs can trigger both extracellular calcium influx and the release from internal stores (Figure 3A). Toxins such as aerolysin and streptolysin O (SLO) have been shown to trigger calcium release from the ER, through the activation of the PLC/IP_3_ pathway, involving G-proteins, which results in the opening of IP_3_Rs in the ER [20,98,107,108]. Other toxins, such as *Clostridium difficile’s* TcdA and TcdB, create pores in endocytic vacuoles, enabling calcium to escape from these compartments into the cytosol [109]. Moreover, *Staphylococcus aureus* leukotoxins such as γ-hemolysin (Hlg) and Panton–Valentine leukocidin (PVL), increase intracellular calcium levels by initially triggering its release from lysosomes, followed by secondary release from the ER [99]. This cascade activates store-operated calcium channels in the plasma membrane. The process is initiated by leukotoxin-induced activation of CD38, a receptor, and NAADP synthase, which mediates calcium release from lysosomal stores via two-pore calcium channels [99]. Interestingly, listeriolysin O (LLO) induces calcium release from the ER via two mechanisms: activation of the PLC/IP_3_ pathway and direct organelle damage to the ER and lysosomes [110]. Although organelle perforation appears to be calcium-independent, the exact mechanism remains unknown.

Overall, this evidence demonstrates that certain PFTs induce passive calcium influx through the activation of alternative signaling pathways, thereby elevating intracellular calcium levels [20]. This suggests that endogenous calcium channels play a critical cooperative role in amplifying and shaping calcium signals beyond initial membrane disruption. Similar mechanisms may work in the context of regulated necrosis, where endogenous channels such as those comprising the PLC/IP_3_R/ORAI1 axis might act as key nodes that integrate and amplify calcium fluxes triggered by membrane pores (Figure 3A). This amplification would ensure a tightly controlled, spatially restricted calcium signal, which is essential for the precise activation of downstream cell death and inflammatory pathways.

One particularly unresolved issue is distinguishing calcium signals generated by regulated channels from those resulting from non-selective membrane pores. It is likely that these two modes of calcium entry differ in amplitude, localization, and duration, but their respective contributions to downstream signaling remain poorly characterized. Moreover, which endogenous channels are primarily involved in the amplification of the calcium signal during regulated necrosis? How do they synergize and coordinate with different PFPs to modulate the amplitude and duration of local signals? Is there a difference in the signaling depending on the nature of the pores? Related to this, how do cells distinguish between lipid- and protein-mediated pores such as those found in ferroptosis and necroptosis or pyroptosis? Do the cells differentiate the signal triggered by extracellular and intracellular pores? How is the local calcium microenvironment regulated to selectively engage specific calcium-binding effectors? Unraveling these questions will enable us to understand the specific role of the calcium code activated during inflammatory forms of cell death.

## 5. Intracellular Events Triggered by Calcium During Necrotic Cell Death

In the previous sections, we have described how calcium is a highly versatile second messenger that facilitates numerous cellular responses through its association with various CaBPs. These proteins transduce calcium signals into different cellular outcomes, including gene expression, cytoskeletal adjustments, membrane repair, and programmed cell death [60,73,111]. In this section, we will discuss current evidence linking calcium signaling with different intracellular processes upon induction of necrotic cell death. We stress the dual role of calcium as a mediator of cell death and other processes modulating immunogenicity, which mostly take place during the sublethal phase of regulated necrosis (Figure 3B).

### 5.1. Calcium as a Promoter of Cell Death

Sustained or overwhelming calcium overload can trigger a cascade of destructive processes. This can involve the activation of calcium-dependent enzymes such as calpains, phospholipases, and nucleases, which degrade the cytoskeleton, disrupt membrane integrity, and initiate cell lysis [12]. Calcium released from the ER or entering from the extracellular space can cause mitochondrial damage, including reactive oxygen species (ROS) production, loss of membrane potential, and release of mitochondrial DNA [70]. Mitochondrial dysfunction is a crucial mechanism by which calcium can promote cell death [70]. Excess cytosolic calcium can be taken up by mitochondria, leading to the opening of the mitochondrial permeability transition pore (MPTP), loss of mitochondrial membrane potential, ATP depletion, and ultimately, necrotic cell death [112]. In agreement with this, pharmacological inhibition of mitochondrial calcium uptake or the MPTP can protect cells from calcium-induced necrosis, highlighting the importance of this pathway [113]. While these processes have been extensively studied in the context of apoptosis [114], the mechanisms by which calcium overload contributes to cytotoxicity during regulated necrosis remain unclear. It is plausible that similar pathways are involved; however, the molecular machinery may differ due to the distinct signaling cascades underlying each form of cell death. Extending previous studies on apoptosis and accidental necrosis [115] may shed light on the specific mechanisms by which calcium induces cytotoxicity in the context of regulated necrosis.

Calcium can also regulate the activation of mediators of regulated necrosis. For instance, calcium released from intracellular stores can trigger pyroptosis, as some evidence shows that inhibition of PLC, the IP_3_R, or SOCE reduces caspase-1 activation [116,117]. Moreover, CaMKII has been identified as a novel substrate of RIPK3 that promotes MPTP opening and mediates necroptosis of myocardial cells through the RIPK3-CaMKII-MPTP signaling pathway [103]. Furthermore, iron–calcium crosstalk in ferroptosis is characterized by a mutually reinforcing relationship: calcium overload increases iron toxicity, and iron-driven ROS production exacerbates calcium dysregulation [118]. This synergy accelerates lipid peroxidation and cell death, making both ions central to the regulation and execution of ferroptosis. Recent research underscored the importance of calcium in the regulation of ninjurin 1 (NINJ1), a protein that plays a crucial role in plasma membrane rupture during various forms of regulated and accidental necrosis [119]. Activation of NINJ1 appears to require transmembrane protein 16F (TMEM16F), a calcium-dependent lipid scramblase, but it remains unclear whether it acts directly on NINJ1 or operates through upstream lipid remodeling [119]. Interestingly, both TMEM16F and NINJ1 have been observed to be important for cell death in ferroptosis and pyroptosis, but not in necroptosis [120,121,122,123].

Taken together, these studies suggest that the pro-death effects of calcium during regulated necrosis may arise from either non-specific or more coordinated mechanisms. On the one hand, calcium may cause cellular damage by disrupting mitochondrial function, organelle organization, or ROS regulation. On the other hand, it may act in a more specific manner by regulating key checkpoints within cell death pathways. Distinguishing between these possibilities is essential to understand the contribution of calcium to regulated necrosis.

### 5.2. Calcium as a Mediator of Membrane Repair

The reasons why calcium can promote cell death in some contexts while counteracting it in others are still unclear. Moderate increases in calcium can activate membrane repair mechanisms, enabling cells to recover from sublethal injury [60]. The first step in initiating repair is sensing loss of membrane integrity. In this sense, the cell can detect the following changes provoked by pore formation: (1) ion imbalance (calcium influx and potassium efflux); (2) loss of transmembrane lipid asymmetry (phosphatidylserine (PS) and sphingomyelin scrambling); and (3) glycan exposure to the cytosol [106,124]. These mechanisms have primarily been studied in the context of membrane injury caused by PFTs, but they may also play a role in the cellular response to membrane damage during regulated necrosis. Next, we review established membrane repair mechanisms, highlighting those already implicated or potentially involved either in PFT-mediated cell death or in regulated necrosis.

If the pores are small in size (less than 1 nm in diameter), the damage can be reversed through spontaneous membrane resealing [124]. To favor membrane fusion, it is essential to reduce membrane tension, which can be achieved mainly by two main processes: remodeling of the apical cytoskeleton and vesicle-mediated membrane fusion. Calcium-dependent proteases, such as calpains, promote membrane repair by cleaving cytoskeletal components and facilitating vesicle fusion [125,126]. In this context, SYT7 acts as a calcium sensor, mediating interactions between vesicles and the plasma membrane [127]. SYT7 also regulates the assembly of soluble N-ethylmaleimide-sensitive-factor attachment protein receptor (SNARE) complexes, which catalyze the fusion of exocytic lysosomes with the wounded plasma membrane and promote repair following calcium influx [128].

When the pores are larger, simple resealing is insufficient, and additional mechanisms are required, such as the removal or internalization of the pores by shedding/endocytosis, or patching [129]. Following SYT7-mediated lysosomal exocytosis, cells undergo a burst of endocytosis to internalize and eliminate the damaged areas. Lysosomal exocytosis releases enzymes such as acid sphingomyelinase (ASM), which hydrolyzes sphingomyelin to generate ceramide at the plasma membrane [130]. Ceramide, with its cone-shaped structure, promotes membrane curvature and invagination, facilitating the endocytosis of injured regions [131]. Inhibition of ASM activity blocks plasma membrane repair, whereas exogenous ASM can restore repair independently of calcium signaling [132]. Furthermore, some pathogens, such as *Neisseria gonorrhoeae* and *Staphylococcus aureus*, exploit ASM-dependent membrane remodeling to invade host cells, highlighting the broader biological significance of these repair pathways [133,134].

In addition to endocytosis-based lesion removal, cells exploit mechanisms involving the ESCRT machinery for outward budding and shedding. Recruited through calcium-sensing proteins such as ALG-2-interacting protein X (ALIX) and programmed cell death 6 (PDCD6), ESCRT-III assembles at wound sites and facilitates membrane scission, shedding damaged portions of the plasma membrane as extracellular vesicles [135]. ESCRT-mediated repair is essential for sealing small wounds in the plasma membrane and in the membranes of lysosomes and pathogen-containing vacuoles (PCVs), as demonstrated during infections with *Salmonella enterica*, *Mycobacterium tuberculosis* (Mtb), and *Coxiella burnetiid* [106]. Interestingly, pathogens such as Mtb have evolved effectors that subvert ESCRT-dependent repair, favoring their escape into the host cytosol [136]. Yet, even in the absence of ESCRT, cells deploy alternative mechanisms in response to PFT-mediated membrane injury. For example, sphingomyelin (SM) exposure on the cytosolic side of damaged membranes can drive repair via neutral SMase-mediated ceramide production. This process promotes inward budding and lesion clearance independently of ESCRT [20]. TMEM16F has also been implicated in promoting the shedding of extracellular vesicles upon plasma membrane damage. This hints at the existence of a sphingolipid-based plasma membrane repair pathway that is analogous to those operating in lysosomes [137]. Unlike ASM-mediated repair, which requires lysosomal exocytosis, TMEM16F-driven pathways might provide a more immediate defense against bacterial toxins and may be less susceptible to hijacking by pathogens [138].

Another mechanism by which cells counteract and repair damage caused by pore formation is membrane clogging and patching [106]. This process relies heavily on the sequential and reversible recruitment of annexins to the plasma membrane, which is guided by their sensitivity to calcium [83]. Annexins with high calcium sensitivity, such as annexins 2 and 6, are first recruited to sites of injury [139]. As calcium levels continue to rise, potentially reaching toxic concentrations, annexins that are less sensitive, such as annexins 1 and 5, are subsequently recruited [139]. Once at the wound site, annexins play specialized roles in supporting membrane repair [82]. Annexin 6 forms an essential repair cap for resealing, while annexin 5 assembles into a protective two-dimensional bandage over the damaged area to stabilize the membrane [140,141]. Annexin 4 promotes membrane closure by inducing curvature at the edges of the wound [142]. Meanwhile, annexin 2 assists in the delivery of dysferlin, which is critical for membrane repair in skeletal muscle. Annexin 7 facilitates the recruitment and assembly of the ESCRT-III complex, participating in the shedding of damaged membrane fragments to restore cellular integrity [143,144].

While these mechanisms have been extensively described in the context of membrane damage mediated by PFTs, little is known about their role in regulated necrosis. In this regard, mounting evidence highlights the shared function of the ESCRT-III machinery in counteracting pyroptosis, necroptosis, and ferroptosis [10,145,146,147]. In pyroptosis and necroptosis, it was shown that the ESCRT-III machinery is rapidly recruited to the plasma membrane at sites where GSDMD and MLKL accumulate, to remove damaged membrane portions [145,146]. This process delays cell lysis and aids cell survival as well as the release of inflammatory cytokines [93]. Notably, the activation of ESCRT-III follows the same kinetics as calcium elevation during ferroptosis and contributes to modulating the inflammatory outcome of this form of regulated necrosis [10]. The involvement of additional membrane repair mechanisms in these forms of cell death is yet to be elucidated. We know that the pores formed by MLKL, gasdermins, or triggered by lipid peroxidation during regulated necrosis differ significantly in size, structure, and dynamics from those formed by bacterial toxins. Nevertheless, they can still induce calcium influx and lipid scrambling, both of which are crucial for the activation of membrane repair. The biophysical properties of these pores will directly influence the calcium signals generated, which can be interpreted by specific CaBPs such as annexins, synaptotagmins, and ALG-2. Therefore, we can expect that, depending on the type and severity of membrane damage, distinct repair pathways will be activated. It is also possible that more than one mechanism will be engaged. These mechanisms could act either synergistically or in a sequential and coordinated manner. Understanding how pore characteristics influence calcium signaling and repair activation is essential for deciphering specific cell fate decisions during different forms of regulated necrosis.

### 5.3. Calcium-Dependent Immunomodulation

Cell death is not the only possible outcome following pore formation, as cells can counterbalance or repair membrane damage [124,129]. The delay between pore formation, calcium elevation, and eventual cell death provides a window during which distinct signaling cascades can shape the molecular signature of dying cells [93]. This signature is influenced not only by the nature of the molecules that are released upon final membrane rupture but also by the signaling events and cellular remodeling processes that occur during the sublethal phase that anticipates cell death (Figure 3B) [93]. As its levels increase during this phase, calcium will take on additional roles, such as modulating immune responses, remodeling the cell surface, and altering the cytoskeleton and cortical architecture, or by regulating gene expression [138].

Following membrane disruption, calcium influx can initiate significant transcriptional changes. Optogenetic systems have been instrumental in elucidating the specific function of membrane damage in signal transduction. They have demonstrated that membrane injury alone is sufficient to trigger these responses, even in cells that ultimately survive the death-inducing event. An integrative study compared the effect of optogenetic activation of MLKL and GSDMD with perforin and digitonin, which act as external membrane stressors. This study demonstrated that the recruitment of S660-phosphorylated PKC to regions of the membrane undergoing calcium influx is a key molecular event that links membrane damage to transcriptional activation. In this context, localized PKC act as a signaling hub, initiating pathways that ultimately lead to changes in cytokine and chemokine production. Specifically, early-response genes, such as *egr1* and *fos*, are activated through MAPK pathways, while cytokines and chemokines such as *cxcl1* and *cxcl10* are upregulated via NF-κB signaling. Notably, this signaling is not shared with ionophores, uncovering the specific role of pore formation in shaping the immune outcome of dying cells [93].

Lipid scrambling has been extensively studied as a calcium-dependent process that is associated with cell death. Indeed, exposure of PS as part of lipid scrambling has long been considered a hallmark of apoptosis. However, recent studies have also observed lipid scrambling in various forms of regulated necrosis. In this context, TMEM16F, a calcium-dependent scramblase, has been shown to contribute to both ferroptosis and pyroptosis [120,121,122]. However, this scramblase appears to be dispensable for necroptosis. Intriguingly, calcium also seems to be unnecessary for PS exposure during necroptosis. This suggests that other scramblases or floppases, which may sense mechanical changes in the membrane, may be involved in this form of regulated necrosis. Another unexplored aspect is the potential contribution of regulated necrosis executioners, such as MLKL and gasdermins, to lipid scrambling. This could occur directly, through the formation of toroidal pores, or indirectly, by facilitating calcium influx into the cytosol or generating membrane perturbations that are sensed by membrane-associated proteins. Understanding the interplay between PFPs and scramblases, as well as the role of calcium in regulating these processes, is important, given that loss of membrane asymmetry regulates other signaling events, such as membrane repair, together with calcium. It may also serve as an ‘eat-me’ signal, marking cells for clearance and influencing downstream immune responses [148].

Exposure of PS is also required for the optimal activation of a disintegrin and metalloproteinase (ADAM)10 and 17, which act as sheddases by cleaving several key immune regulators, such as TNF and the IL-6 receptor (IL-6R), thereby influencing immune responses [149,150,151]. Notably, ADAM17 is activated in the early stages of necroptosis, contributing to inflammation and cell migration [152]. Although membrane shedding occurs prior to extensive membrane rupture during necroptosis, it is closely associated with lytic cell death. Indeed, TNF shedding is also induced by PFTs. This suggests that the activation of ADAM proteases is driven by changes in the biophysical properties of the membranes, and could play a role in other forms of regulated necrosis [153]. Together, the proteolytic events orchestrated by ADAM10/17 following TMEM16F activation would finely tune the balance between immune activation, tolerance, and inflammation during regulated necrosis.

Overall, this emerging evidence suggests that calcium plays a key immunomodulatory role in regulated necrosis, probably influencing the balance between immune activation and resolution in inflamed tissues. While some calcium-dependent responses, such as the induction of cytokines and chemokines via PKC activation, are better understood, many processes, such as plasma membrane and cytoskeleton remodeling, remain poorly explored. Future research should focus on identifying the CaBPs that sense membrane damage and on elucidating the downstream signaling pathways that are activated. This will help identify common and unique calcium-dependent checkpoints that influence the inflammatory outcomes of regulated necrosis. The specificity of these responses should be tightly controlled by the amplitude, duration, and spatial localization of calcium signals, as well as the distinct repertoire of CaBPs expressed in different cell types.

## 6. The Toolbox for Studying Calcium Signaling: Towards Its Application to Regulated Necrosis

Tracking and elucidating the precise role of calcium during cell death remains challenging due to the intricate and multifaceted networks that it orchestrates. Recent advancements in the development of experimental techniques and tools, including high-resolution imaging, calcium-sensitive probes, genetically encoded calcium indicators, OMICS, and single-cell analysis, have opened new avenues for unraveling these complex regulatory mechanisms with unprecedented precision. In this section, we summarize recent technological advances in these techniques and discuss how they can be used to improve our understanding of the molecular mechanisms underlying calcium signaling during regulated necrosis.

### 6.1. The Broad Repertoire of Calcium Indicators

Calcium-sensitive fluorescent dyes and genetically encoded calcium indicators (GECIs) are fundamental to the characterization of intracellular calcium signaling, as they facilitate the qualitative and quantitative analysis of calcium in the cytoplasm and sub-cellular organelles, including the plasma membrane, the ER, and mitochondria. Significant improvements in both chemical and genetically encoded indicators [154,155] make it difficult to select the optimal probe. Here, we first provide an overview of the calcium indicators that are currently available and compare their respective advantages and disadvantages to help select the most suitable indicator for studying cell death (Table 2).

**Figure 4:**
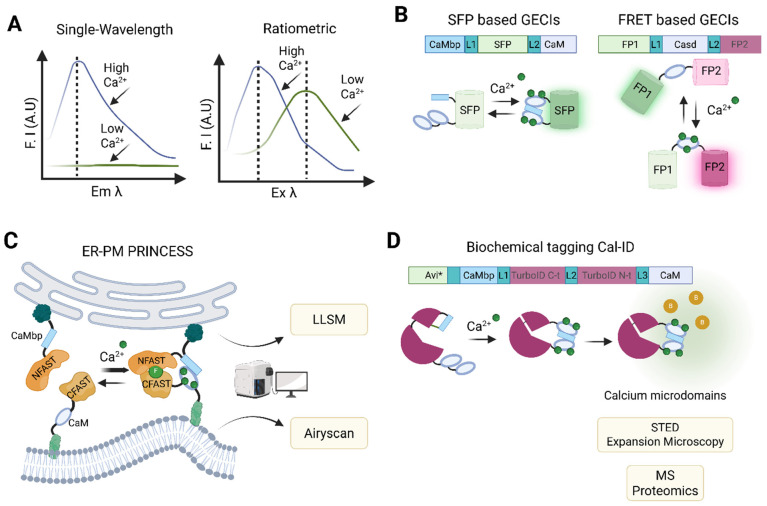
Calcium indicators and probes and their applications. (**A**) Representative spectral characteristics of single-wavelength (left panel) and ratiometric (right panel) chemical dyes. (**B**) Schematic representation of single fluorescent protein (upper panel) and FRET (lower panel) based GECIs. (**C**) ER-PM PRINCESS design. PRINCESS integrates calcium-sensing modules into splitFAST system, enabling simultaneous detection of MCS morphology and associated calcium dynamics within a single probe. It can be applied to advanced microcopy methods such as LLSM and Airyscan microscopy. (**D**) Principle of Cal-ID system. This BioID-based construct switches conformation between inactive and active states depending on its calcium-binding status, promoting the biotinylation, through avidin (Avi*)-biotin bond, of nearby proteins located at calcium microdomains. Cal-ID can be combined with advanced super-resolution microscopy and proteomics to characterize the localization and molecular composition of calcium microdomains. F.I.: fluorescence intensity, A.U.: arbitrary units, Em: emission, Ex: excitation, SFP: single fluorescent protein, GECIs: genetically encoded calcium indicators, FRET: Förster or fluorescence resonance energy transfer, CaM: calmodulin, CaMbp: CaM binding protein, L: linker, FP: fluorescent protein (FP), Casd: calcium sensing domain, ER: endoplasmic reticulum, PM: plasma membrane, PRINCESS: PRobe for INterorganelle calcium-Exchange Sites, MCS: membrane contact site, splitFAST: split fluorescence-activating and absorption-shifting tag (C or N-terminal), LLSM: lattice light-sheet microscopy, Cal-ID: calcium-dependent BioID, STED: stimulated emission depletion, MS: mass spectrometry. Created with Biorender.com.

Calcium-sensitive fluorescent dyes are currently the most versatile and widely used tools for analyzing cellular calcium responses in different experimental settings, including cell death (Figure 4A). The development of membrane-permeable acetoxymethyl ester (AM) derivatives of the chemical calcium indicators, such as Fura-2, Fluo-4 and Rhod-2, coincided with the increasing accessibility of confocal fluorescence microscopy. This combination has led to a significant increase in the number of studies investigating intracellular calcium signaling. Subsequently, calcium indicators with enhanced response amplitudes, diverse spectral properties, and calcium binding affinities were developed, including probes capable of membrane tethering or selective accumulation in intracellular organelles [157,159,172]. Despite requiring ultraviolet excitation, early-generation dyes such as Fura-2 remain indispensable for calibrated measurements in neurons and immune cells [173,174,175]. Recent advances have addressed the limitations of the earlier dyes. For instance, Cal-590-AM, which operates in the red spectrum, provides a higher signal-to-noise ratio and avoids mitochondrial accumulation [158]. Similarly, Cal-520-AM offers improved intracellular retention and sensitivity compared to Fluo-4-AM or Oregon Green BAPTA-1-AM (OGB-1-AM) [156]. This progression reflects a continuous optimization of dyes to balance quantitative accuracy, spectral compatibility, and application-specific needs.

Currently, two main types of synthetic dye are used to measure intracellular calcium levels. These are categorized according to their spectral properties (Figure 4A). Single-wavelength indicators, such as Fluo-4-AM and Cal-520-AM, exhibit substantial changes in fluorescence intensity upon calcium binding without shifting their excitation or emission wavelengths. This reduces spectral overlap in multiplexed experiments [159]. In contrast, ratiometric dyes, such as Fura-2-AM and Indo-1-AM, undergo shifts in their emission or excitation spectra upon calcium binding. This enables absolute quantification of calcium concentrations via fluorescence ratios, albeit requiring broader spectral ranges [176]. Dye selection also depends on calcium-binding affinity. High-affinity dyes (e.g., Fura-2) are suitable for detecting low cytosolic calcium levels, whereas low-affinity ones (e.g., Rhod-2) are better suited to measuring high calcium concentrations in organelles such as the ER [159]. Despite their widespread use, fluorescent calcium indicator dyes have several limitations that restrict their use in certain experimental settings. One major drawback stems from their non-proteinaceous nature, which hinders precise subcellular targeting [162]. To address this issue, targeted esterase-induced dye loading (TED) has been employed to improve the delivery of low-affinity indicators into the ER lumen [174,177]. Advances in synthetic biology, such as the use of split esterases, offer the potential to improve TED specificity and efficacy [178,179]. However, implementing these systems requires the introduction of orthogonal enzyme–substrate pairs via expression vectors, such as the lentiviral constructs used to deliver carboxyesterases. This adds complexity and limits applicability in certain models [177].

Calcium-sensitive dyes have been widely used to study the role of calcium signaling in cell death. For instance, Fluo-4 AM and its enhanced derivative, Fluo-8 AM, have been frequently used to monitor intracellular calcium dynamics during pyroptosis, necroptosis, and ferroptosis [89,115,180]. Ratiometric dyes such as Fura-2 AM have provided more quantitative measurements by compensating for variability in dye loading and photobleaching [181,182]. All these dyes are commonly used in flow cytometry and confocal microscopy studies, often in combination with cell death markers. This has enabled the real-time tracking of calcium fluctuations associated with regulated necrosis and their relationship with other hallmarks of cell death at population and single-cell levels [10]. However, these dyes also have several limitations, including quenching issues, phototoxicity, limited subcellular resolution, and difficulties with long-term imaging in live cells. In addition to targeting issues, chemical calcium indicators can disrupt cellular physiology. For example, AM ester-based derivatives such as Fluo-4 AM have been shown to inhibit Na^+^/K^+^ ATPase activity. This disrupts ionic homeostasis, impairs cellular metabolism, and increases extracellular potassium concentrations, all factors that can affect experimental interpretation [183]. Furthermore, loading cells with these dyes in bulk often prevents capturing the spatiotemporal complexity of intracellular calcium signaling. For example, this interferes with the ability to resolve localized calcium microdomains, such as those formed near calcium channels [184,185,186]. These limitations highlight the need for indicators with superior specificity and dynamic range that can be targeted to specific subcellular compartments [187].

Genetically encoded calcium indicators (GECIs) are sensors based on fluorescent proteins (FPs) that have been engineered by fusing calcium-binding domains to them [188,189]. They have been shown to outperform chemical indicators [190]. The effectiveness of these indicators is determined by the properties of both the fluorescent protein and the calcium-binding domain. Most GECIs use either CaM or troponin C (TnC) [14,188]. These domains confer fast kinetics and a broad dynamic range, making them well-suited for capturing rapid changes in intracellular calcium levels. The GCaMP family (Table 2), a widely used class of GECIs, is based on a circularly permuted green fluorescent protein (cpGFP) fused to CaM and the M13 peptide from myosin light chain kinase. Upon calcium binding, CaM undergoes a conformational change that brings M13 into close proximity, thereby increasing fluorescence intensity in a calcium-dependent manner (Figure 4B). Despite their utility, these calcium-binding domains can exhibit unintended biological activity, potentially interfering with native signaling pathways in mammalian cells [191,192]. Additionally, GECIs function as exogenous calcium buffers, particularly those with high affinity and multiple binding sites, which can significantly alter the endogenous calcium buffering capacity of the cell. As this buffering effect can distort physiological calcium dynamics, careful control of expression levels is essential when using GECIs.

In general, GECIs can be classified as single-wavelength GECIs, consisting of a calcium-sensing domain and a single fluorescent protein, whose fluorescence intensity changes when shifting between Ca^2+^-free and Ca^2+^-bound states. Alternatively, they can be classified as Förster or fluorescence resonance energy transfer (FRET) GECIs (Figure 4B), consisting of two fluorescent proteins linked by a calcium-binding domain [193,194,195]. FRET-based sensors are the preferred option for quantifying absolute calcium concentrations, as they rely on changes in energy transfer between fluorophores upon calcium binding. In contrast, single-wavelength non-ratiometric GECIs (e.g., GCaMP6 variants, jGCaMP7/8, RCaMP, and jRGECO1) are ideal for tracking calcium dynamics, as they require only one detection channel and allow multiplexing with other fluorescent probes [165,188,196,197,198].

GECIs, such as GCaMP, have emerged as powerful tools for studying calcium signaling in the context of regulated necrosis. These probes have been successfully used to monitor calcium dynamics during necroptosis [92,164], pyroptosis [88], and ferroptosis [199], as well as during MLKL-mediated membrane damage in plant systems [200]. Compared to chemical dyes such as Fluo-4AM, GCaMP indicators offer superior photostability and enable long-term imaging with minimal phototoxicity. Furthermore, they enable high spatial and temporal resolution and can be targeted to specific subcellular compartments. Despite these advantages, a key limitation is the requirement for genetic manipulation, either through transient transfection or the generation of stable cell lines, which may not be feasible in all experimental systems. Nevertheless, due to their sensitivity, reduced signal variability, and compatibility with live-cell imaging, GCaMPs are increasingly considered the preferred choice for studying calcium dynamics during regulated cell death.

### 6.2. Application of GECIs to Track Intracellular Calcium Dynamics

Both the ER and mitochondria are central to intracellular calcium regulation and cell death, yet targeting these organelles to track calcium dynamics has been challenging. Early generation GECIs, such as GFP-based FRET sensors and aequorin (Table 2), addressed the targeting limitations of small-molecule indicators [201,202,203,204] by incorporating organelle-specific tags, enabling localized calcium measurements. Nevertheless, these GECIs often suffered from drawbacks, including spectral overlap, low brightness, or limited compatibility with multiplexed imaging [171]. To overcome these limitations, different calcium-measuring organelle-entrapped protein indicators (CEPIAs) were developed [171]. Based on single-FP-cytosolic GECIs (GECOs), CEPIAs have unique features. They offer enhanced spatiotemporal resolution, improved dynamic range, and organellar specificity. They are available in green (G-CEPIA), red (R-CEPIA), and ratiometric (GEM-CEPIA) variants that allow the simultaneous imaging of ER and mitochondrial calcium with subcellular precision. Furthermore, their high signal-to-noise ratio and organelle-specific targeting have revealed heterogeneous mitochondrial calcium responses and ER–mitochondrial crosstalk. Overall, CEPIAs represent a significant step forward in GECI technology, providing essential tools for investigating organelle interactions in both physiological and pathological contexts [170]. Although CEPIA probes show great promise in the study of calcium flux dynamics across organelles during regulated necrosis, several challenges must be considered (Table 2). CEPIA imaging may lack the necessary spatiotemporal resolution to capture calcium transfer across organelles and the cytosol, as these processes are highly dynamic and localized. Furthermore, mislocalization or overexpression of the probes can disrupt organelle function and calcium homeostasis, thereby affecting cell death pathways [205,206]. Therefore, CEPIA data should be validated using complementary methods, such as proximity labeling, to gain a more complete and accurate understanding of the involvement of the ER and mitochondria in regulated necrosis.

Studying membrane contact sites (MCSs), which are critical hubs for organelle communication and calcium transfer, has also remained challenging due to their dynamic nature, particularly in the context of cell death. Recently, the PRobe for INterorganelle calcium-Exchange Sites (PRINCESS) family of probes was developed, overcoming some of the limitations of existing calcium probes [206]. This innovative biosensor integrates calcium-sensing modules (e.g., GCaMP) into the split fluorescence-activating and absorption-shifting tag (splitFAST) system. It enables simultaneous detection of MCS morphology and associated calcium dynamics within a single probe (Figure 4C). splitFAST is a chemogenetic reporter that uses two fragments (e.g., NFAST and CFAST), which assemble and fluoresce only at organelle proximity sites (e.g., ER–mitochondria or ER–plasma membrane). This enables rapid (<1 min), reversible detection of MCSs in live cells [207]. PRINCESS outperforms existing probes due to its ability to rapidly and reversibly detect MCSs and its compatibility with other sensors (e.g., ROS and pH) [206]. This combination of chemogenetic MCS reporters and advanced GECIs marked a paradigm shift in organelle interaction studies, by integrating spatial precision with functional readouts. The ability of PRINCESS to detect the formation of MCSs and the associated calcium fluxes simultaneously, especially within the relevant range for cell death, makes it uniquely suited to dissecting how axes, such as those coordinated at the plasma membrane–ER or ER–mitochondria contact sites, integrate into pro-death signaling events. This dual capability would provide a direct link between calcium microdomain activity at MCSs and the initiation and propagation of cell death pathways. However, challenges remain in distinguishing between closely spaced or overlapping MCSs and capturing the full complexity of calcium-dependent signaling cascades that drive diverse forms of cell death.

### 6.3. Emerging Microscopy and OMICs Tools to Study Calcium Localization and Dynamics in Cells

Although traditional fluorescence microscopy has played a key role in the study of calcium dynamics, its diffraction-limited resolution (~200 nm) prevents visualization of subcellular calcium signals that occur within tightly confined microdomains. The introduction of advanced and super-resolution microscopy has transformed this field by enabling nanoscale spatial resolution and thus uncovering calcium signaling events that were previously inaccessible, with greater precision. Methods such as lattice light-sheet microscopy (LLSM) [206], Airyscan [208,209], stimulated emission depletion (STED) microscopy [210,211], and expansion microscopy [209,212], especially when combined with genetically encoded calcium indicators, have enabled the detailed, dynamic tracking of calcium fluxes in both living cells and fixed samples. Their application has allowed resolving local calcium microdomains with nanometer-scale detail, tracking dynamic calcium changes in and between organelles, and mapping the spatiotemporal architecture of calcium signaling pathways during physiological and pathological events. They therefore hold significant potential for studying calcium dynamics during regulated necrosis. Despite this promise, several challenges remain. Super-resolution methods can cause phototoxicity and photobleaching, which limit the ability to conduct long-term live-cell imaging of dynamic cell death processes [213,214]. Moreover, the nanometer scale and highly transient nature of calcium microdomains require extremely sensitive and rapid imaging, which remains technically challenging.

The recent introduction of a proximity labeling-based BioID system, engineered to switch conformations between inactive and active states depending on its calcium-binding status, opened a unique opportunity to the study of calcium microdomains. This calcium-dependent BioID (Cal-ID) biotinylates nearby proteins when local calcium levels are elevated (Figure 4D). Since biotinylated proteins can be visualized using super-resolution microscopy methods and identified by mass spectrometry (MS), this approach can provide a biochemical record of calcium microdomains, with high spatial resolution. Importantly, as Cal-ID can be targeted to different cellular locations, it enables the study of localized calcium signaling events [209]. We can envision that targeting Cal-ID to specific organelles, such as the plasma membrane, the ER, or the mitochondria, would allow mapping the specific localization of these microdomains during regulated necrosis. Moreover, it would enable tracking changes in their molecular composition, facilitating the identification of key hotspots involved in the initiation and propagation of downstream signaling.

Finally, the application of OMICS methods (including genomics, proteomics, and transcriptomics) holds promise for the study of calcium signaling during regulated necrosis, as these methods provide a comprehensive, systems-level understanding of these cellular processes. In this regard, a pioneering study used mRNA sequencing (mRNA-seq) to profile mRNA expression following the induction of sublethal plasma membrane disruption associated with various forms of necrotic cell death [93]. The analysis revealed the activation of a unique pattern of gene expression for specific chemokines and cytokines, which was corroborated by qPCR and ELISA. By comparing calcium-containing and calcium-free media conditions, it was possible to determine that this response depended on extracellular calcium. This study paved the way for the application of other OMICS methods, such as proteomics and phosphoproteomics, to identify further calcium-dependent proteins and signaling cascades that mediate regulated necrosis [93].

## 7. Concluding Remarks and Outlooks

The mediators of regulated necrosis and external PFTs converge on a shared mechanism involving the disruption of plasma membrane integrity and the increase in cytosolic calcium to influence cell fate. However, the exact mechanisms by which plasma membrane damage initiates and propagates calcium signaling remain to be elucidated. Is pore formation required for this response, or are earlier events such as protein binding to membranes and oligomerization sufficient to initiate it? In addition to the influx of calcium from the extracellular media, this ion can be released from intracellular stores, primarily via channels in the ER and mitochondria. Which channels are activated, and do they act synergistically with membrane pores to further amplify the signaling cascades triggered by membrane damage? It is unclear what triggers the activation of these channels. One possibility is that they are regulated by calcium, changes in osmotic pressure, or less drastic changes in the biophysical properties of the membrane, such as alterations in membrane tension or lipid scrambling.

The precise mechanisms by which calcium influx dictates cell fate, tipping the balance between repair and death, remain unclear. Further research is needed to unravel how different calcium channels, CaBPs, and downstream effectors such as ESCRT-III, additional components of the membrane repair machinery, and NINJ1 are integrated into these processes. How does calcium signaling contribute to shaping the immunogenicity of regulated necrosis? Identifying parallels with cellular responses triggered by PFTs could provide valuable insights into these fundamental mechanisms. In this context, it is critical to identify and characterize the specific functions of diverse CaBPs involved in these pathways. Gaining mechanistic insight into the shared hubs in the calcium signaling and regulated necrosis networks, as well as their versatility across cell types and tissues, could open further avenues for treating inflammatory diseases associated with these forms of cell death.

The recent and rapid development of more sophisticated calcium biosensors has enabled the high-resolution and high-throughput analysis of calcium dynamics. Applying this toolkit to the study of long-term and heterogeneous calcium events connected to regulated necrosis is essential for understanding the spatial and temporal complexity of these intricate network of the cell.

## Figures and Tables

**Table 1:** Endogenous calcium channels involved in regulated necrosis and PFT-induced cell death.

Type of Cell Death	Receptor/Channel	Localization in Cell	Contribution to Cell Death	References
Pyroptosis	IP_3_R2	ER	Releases Ca^2+^ from ER, promoting NLRP3/caspase-1/GSDMD activation.	[89]
	CaSR/GPRC6A	Plasma membrane (GPCRs)	Sense extracellular Ca^2+^; activate NLRP3 inflammasome via PLC/IP_3_ signaling.	[90]
	PLCγ1/IP_3_R	Cytosol/ER	Enhance Ca^2+^ signaling and promotes GSDMD-N translocation to membrane.	[91]
Necroptosis	TRPM7	Plasma membrane	May facilitate Ca^2+^ entry following MLKL activation.	[92]
	STIM1/ORAI1	ER (STIM1), Plasma membrane (ORAI1)	Mediate SOCE; sustain intracellular Ca^2+^ after membrane permeabilization.	[93]
Ferroptosis	VDAC	Outer mitochondrial membrane	Involved in early Ca^2+^ flux (first wave, with erastin-1).	[94]
	IP_3_R1	ER	Mediates ER Ca^2+^ release; required for RSL3-induced ferroptosis.	[95]
	MICU1	Mitochondria	Regulates mitochondrial Ca^2+^ uptake; necessary for lipid peroxidation.	[96]
	ORAI1/ORAI3	Plasma membrane	Involved in SOCE; their inhibition reduces ferroptotic death.	[97]
PFT-Induced Death	IP_3_Rs	ER	Activated via PLC; mediate Ca^2+^ release from ER.	[98]
	TPCs	Lysosomes	Mediate Ca^2+^ release after NAADP signaling (triggered by leukotoxins).	[99]
	CD38	Lysosomes	Activates NAADP synthesis; initiates lysosomal Ca^2+^ release via TPCs.	[99]

**Table 2:** General characteristics of commonly used calcium indicators.

Calcium Indicator		Mechanism	Advantages	Limitations	Examples	Ref.
Chemical indicators	Single-Wavelength (Intensity-Based) Indicators	Show an increase in fluorescence intensity upon calcium binding without changing excitation/emission wavelengths.	-Easy to use with standard fluorescence microscopes. -Suitable for high-throughput and multiplexed assays due to limited spectral overlap.	-Signal depends on dye concentration and loading efficiency. -Not suitable for absolute calcium quantification.	Fluo-4-AM, Cal-520-AM, Rhod-2-AM, OGB-1-AM, Cal-590-AM	[156,157,158]
	Ratiometric Indicators	Exhibit a shift in excitation or emission spectra upon calcium binding, allowing the ratio of two wavelengths to be used for quantification.	-Allow absolute measurement of calcium. -Compensate for uneven dye loading, photobleaching, and cell thickness.	-Require more complex optics and broader spectral range. -Some require UV excitation, which can cause phototoxicity.	Fura-2-AM, Indo-1-AM	[157,159,160]
	Targeted-Esterase Induced Dye Loading (TED) Dyes	Use of engineered esterases to hydrolyze AM esters in specific compartments.	-Enable compartment-specific dye localization.	-Require expression of exogenous enzymes (e.g., via viral vectors). -Share some drawbacks with synthetic dyes.	Low-affinity AM dyes: Fluo-5N-AM, Mag-Fluo4-AM, Mag-Fura2-AM with targeted carboxylesterases	[159,161,162]
Genetically encoded calcium indicators (GECIs)	Single-Fluorescent Protein (Single-FP) Indicators	Conformational changes in a single FP upon calcium binding alter fluorescence intensity.	-High dynamic range. -Cell-type-specific targeting via promoters. -Compatible with in vivo two-photon microscopy.	-Green variants suffer from tissue scattering/blood absorption. -Photobleaching in prolonged imaging.	GCaMP6 variants, jRGECO1, FR-GECIs	[163,164,165]
	FRET-Based (Ratiometric) Indicators	Calcium binding alters FRET between two FPs.	-Ratiometric measurements correct for dye concentration/bleaching. -Suitable for absolute calcium quantification.	-Lower dynamic range. -Spectral overlap complicates multiplexing.	Cameleon (YCam): YC3.6, TN-XXL Twitch	[166,167]
	Bioluminescent Indicators	Calcium-dependent light emission without external illumination.	-No phototoxicity or autofluorescence. -Ideal for long-term imaging in sensitive tissues.	-Low light output, requires sensitive detectors. -Non-reversible, consumes substrate.	Aequorin, Nano-lantern	[168,169]
	Organelle- Targeted GECIs	GECIs engineered to monitor calcium dynamics within specific intracellular compartments.	-Precision avoid cytosolic contamination. -More stable than ester-loaded dyes. -Organelle-specific tags ensure accurate localization.	-High-affinity probes (e.g., GCaMP6) may saturate in high-calcium organelles like the ER. -Ratiometric CEPIA (GEM) requires pH controls in acidic compartments. -Overexpression artifacts may alternative calcium handling.	ER: ER-GCaMP6, CEPIA1er Mitochondria: 4mtGCaMP6f, Mitycam, 4mtCEPIA Nucleus: NLS-GCaMP6	[170,171]

## Data Availability

Not applicable.
